# YY1-Targeted RBM15B Promotes Hepatocellular Carcinoma Cell Proliferation and Sorafenib Resistance by Promoting TRAM2 Expression in an m6A-Dependent Manner

**DOI:** 10.3389/fonc.2022.873020

**Published:** 2022-04-14

**Authors:** Chunzhong Tan, Peng Xia, Hao Zhang, Kequan Xu, Pengpeng Liu, Deliang Guo, Zhisu Liu

**Affiliations:** ^1^ Department of Hepatobiliary & Pancreatic Surgery, Zhongnan Hospital of Wuhan University, Wuhan, China; ^2^ Department of Translational Medicine Research Center, Wuhan University, Wuhan, China

**Keywords:** N6-methyladenosine, mRNA stability, RBM15B, TRAM2, hepatocellular carcinoma

## Abstract

As one of the most common internal modifications in eukaryotic mRNA, N6-methyladenosine (m6A) modification is involved in the pathogenesis of many diseases, including hepatocellular carcinoma (HCC). In this study, we explored the prognostic significance of the expression of RNA binding motif protein 15B (RBM15B) in HCC, by studying specimens collected from clinical subjects. RBM15B is highly expressed in HCC patients and indicates a poor prognosis. Functionally, overexpression of RBM15B promotes HCC cell proliferation and invasion and induces sorafenib resistance in HCC cells. Mechanistically, we confirmed that RBM15B is transcriptionally activated by YY1 and regulates the stability of TRAM2 mRNA in an m6A-dependent manner. Overall, our results reveal a YY1-RBM15B-TRAM2 regulatory axis and highlight the critical role of RBM15B and m6A modifications in HCC. These findings may provide a novel mechanism and therapeutic targets for the treatment of HCC.

## Introduction

Hepatocellular carcinoma (HCC) is the sixth most common cancer globally and the fourth leading cause of cancer-related death globally (1, 2). Although studies in recent years have led to improvements in the diagnosis rate and treatment options for HCC, the overall 5-year survival rate of HCC patients is still less than 40% due to the lack of effective treatments (3). Given the grim outlook of HCC, clarification of the mechanisms involved in HCC progression is essential to facilitate the development of effective novel therapeutic targets.

In recent years, the development of RNA epitranscriptomics has attracted increasing attention due to its vital role. To date, more than 150 chemical modifications have been identified in protein-coding and noncoding RNAs (4). N6-methyladenosine (m6A) is the most abundant internal modification of eukaryotic mRNA and has been shown to play a vital role in various biological processes, such as tissue regeneration, cell proliferation, embryonic growth, and stem cell differentiation (5). m6A modification is a reversible RNA modification process and is dynamically regulated by methyltransferase complexes (m6A writers), demethylases (m6A erasers), and m6A-binding proteins (m6A readers) (6, 7). It is a determinant of cytoplasmic mRNA stability and affects the stability, splicing and translation of specific mRNAs (8). However, recent studies have shown that m6A modification is related to tumor differentiation, tumorigenesis, proliferation and invasion and to both proto-oncogenes and anti-oncogenes in malignant tumors (6, 8).

RNA binding motif protein 15B (RBM15B), a component of the methyltransferase complex (MTC), can bind the METTL3 and WTAP proteins and recruit them to specific sites in RNA (9, 10). In this study, we demonstrated for the first time that the expression level of RBM15B is significantly upregulated in HCC and that upregulated RBM15B increases the overall m6A methylation level of HCC. RBM15B significantly promotes HCC cell proliferation and metastasis *in vivo* and *in vitro* and induces sorafenib resistance. Mechanistically, YY1 activates the transcription of RBM15B, and RBM15B inhibits the degradation of TRAM2 mRNA through an m6A-dependent mechanism, thereby promoting the progression of HCC. Our study identified RBM15B as a new oncogenic molecule in HCC and proved that the YY1-RBM15B-TRAM2 signaling pathway might be a new mechanism regulating the occurrence and progression of HCC.

## Results

### RBM15B Is Overexpressed in HCC and Associated With a Poor Prognosis in HCC Patients

**Figure 1:**
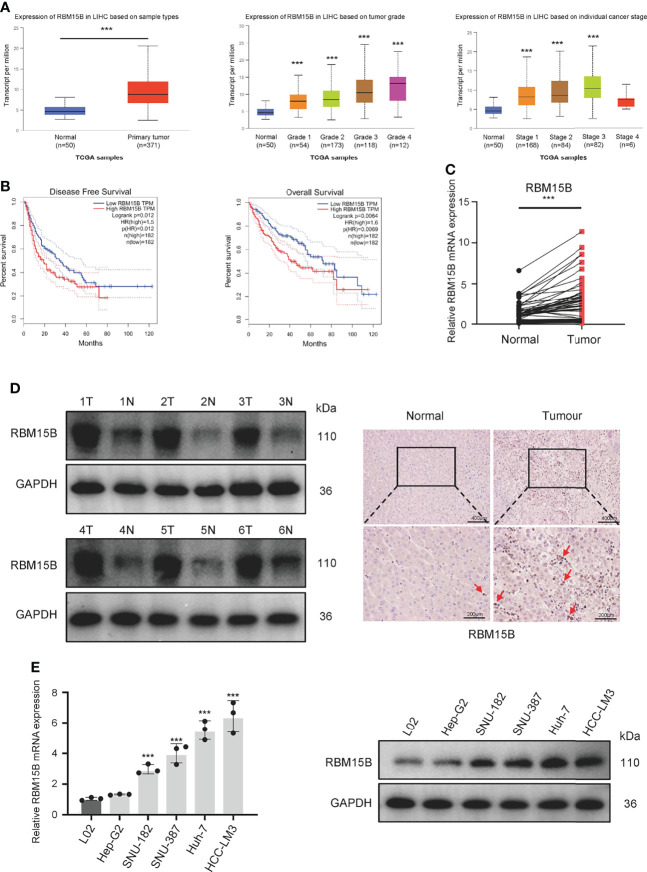
RBM15B expression is significantly increased in HCC. **(A)** Analysis of RBM15B mRNA expression according to the TCGA database.**(B)** Overall and relapse-free survival analysis of RBM15B according to the TCGA database. **(C, D)** Representative RBM15B mRNA and protein expression in 80 pairs of tumor tissues. **(E)** RBM15B mRNA and protein expression in the L02 and 6 HCC cell lines. ***p < 0.001.

Analysis of The Cancer Genome Atlas (TCGA) database showed that the transcription level of RBM15B was significantly upregulated in HCC tissues compared with normal liver tissues and was significantly positively correlated with the pathological stage and grade of HCC (**Figure 1A**). Kaplan-Meier analysis showed that high levels of RBM15B expression were associated with worse survival in HCC patients (**Figure 1B**). Moreover, the TCGA database and the Cancer Cell Line Encyclopedia (CCLE) database showed that RBM15B was overexpressed in most tumor tissues and cell lines (**Figures S1A, B**). In addition, we examined the expression of RBM15B in 80 pairs of HCC tissues and corresponding nontumor tissues. qRT-qPCR, Western blotting and immunohistochemistry (IHC) assays showed that the mRNA and protein levels of RBM15B in HCC tissues were significantly upregulated compared with those in the corresponding nontumor tissues (**Figures 1C, D**). Subsequently, we further analysed the relationship between RBM15B expression and the clinicopathological characteristics of HCC patients, and the results showed that RBM15B expression was significantly correlated with tumor size (P=0.0111), tumour-node-metastasis (TNM) stage (P=0.0436) and portal vein tumor thrombosis (PVTT) stage (P=0.0070) (**Table 1**). Finally, we evaluated the expression of RBM15B in a normal liver cell line (L02) and six different HCC cell lines. The mRNA and protein levels of RBM15B in HCC cell lines (SNU182, SNU387, Huh-7, HCC-L3M) were significantly higher than those in the L02 cell line (**Figure 1E**).

**Table 1:** Correlation between clinicopathological features and RBM15B expression in HCC tumor tissues.

Characteristics	Number	RBM15B expression		P value
		High	Low	
Gender				0.6416
Male	43	22	21	
Female	37	17	20	
Age				0.8161
<65	51	25	26	
≥65	29	15	14	
HBV infection				0.6020
No	21	10	11	
Yes	59	32	27	
AFP				0.4586
≤400 (μg/L)	23	10	13	
>400 (μg/L)	57	30	27	
Tumour size				0.0111^*^
≤5cm	42	18	24	
>5cm	38	27	11	
TNM staging				0.0436^*^
I-II	43	17	26	
III-IV	37	23	14	
Lymph node metastasis				0.3291
Absent	56	26	30	
Present	24	14	10	
BCLC stage				0.1227
Low	53	24	29	
High	27	16	9	
PVTT				0.0070^*^
NO	36	12	24	
YES	44	28	16	

*The expression of RBM15B were compared between the tumor tissue and the normal tissue. BCLC, Barcelona Clinic Liver Cancer; PVTT, portal vein tumor thrombus. Italics indicate statistically significant values. *P < 0.05.

### RBM15B Promotes the Proliferation and Invasion of HCC

**Figure 2:**
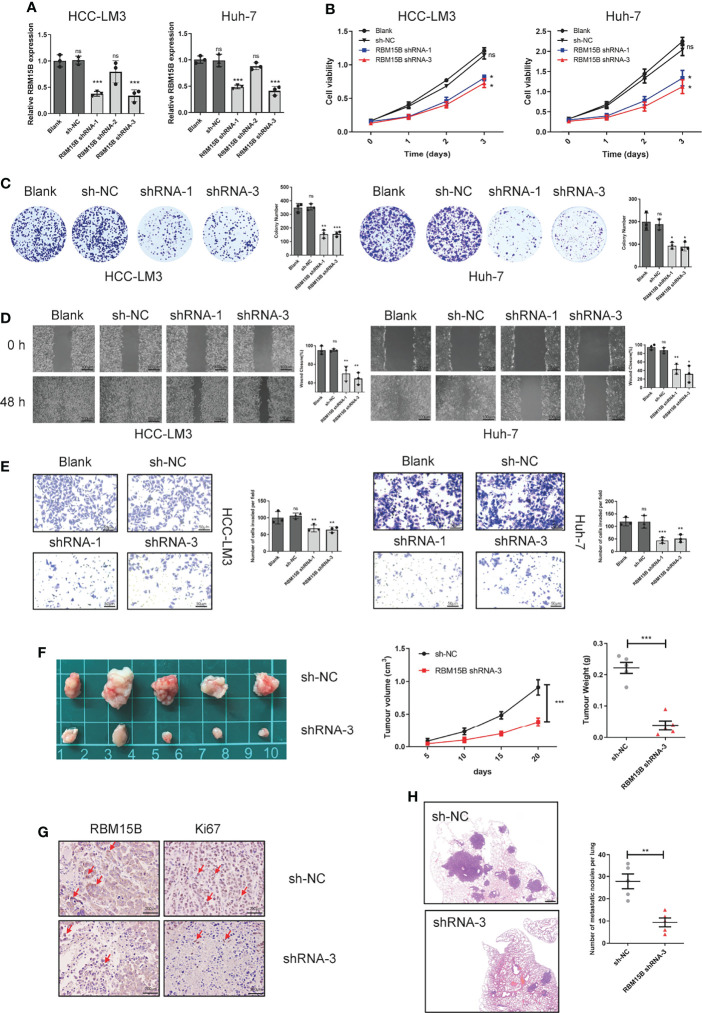
RBM15B promotes HCC cell proliferation and metastasis. **(A)** The expression of RBM15B in HCC cell lines with shRNA-mediated knockdown. **(B, C)** CCK-8 and colony formation assays showing the proliferation ability of HCC cell lines (n=6). **(D, E)** Scratch wound healing motility assay showing cell migration in HCC cell lines (n=3). **(E)** Transwell Matrigel invasion assay showing cell invasion in HCC cell lines (n=3). **(F)** Tumor growth curves of xenograft models established from control or stable RBM15B knockdown HCC-LM3 cells (n=5). **(G)** RBM15B and Ki-67 expression was determined by IHC analysis. **(H)** Metastasis in the lungs of nude mice injected with control or stable RBM15B knockdown HCC cells (n=5). *p < 0.05; **p < 0.01; ***p < 0.001. ns means no significant difference.

To clarify the role of RBM15B in regulating the progression of HCC, we selected HCC cell lines HCC-LM3 and Huh-7 with relatively high RBM15B levels to stably knockdown RBM15B expression and selected the HCC cell line Hep-G2, which has a relatively low RBM15B level, to overexpress RBM15B (**Figures 2A**, **S2A**). The results of Cell Counting Kit-8 (CCK-8) and colony formation experiments showed that RBM15B knockdown inhibited the proliferation of HCC cells (**Figures 2B, C**), while RBM15B overexpression had the opposite effect (**Figures S2B, C**). In addition, scratch wound healing motility assays and Transwell invasion assays showed that RBM15B knockdown significantly reduced cell invasion and migration (**Figures 2D, E**), while RBM15B overexpression led to the opposite effect (**Figures S2D, E**). To further evaluate the effect of RBM15B on tumor growth and metastasis *in vivo*, nude mouse models of subcutaneous tumor formation and tail vein lung metastasis were established using the HCC-LM3 cell line. Our results showed that knocking down RBM15B inhibited tumor growth *in vivo* (**Figure 2F**). The IHC results showed that after knocking down RBM15B, the proliferation-related protein Ki-67 was significantly downregulated (**Figure 2G**). The tail vein lung metastasis model results showed that after knocking down RBM15B, the number of lung metastatic nodules decreased significantly (**Figure 2H**). Together, these findings indicate that RBM15B is critically involved in the proliferation and metastasis of HCC cells. It has been reported that cell cycle changes and EMT progression play a vital role in tumor proliferation and metastasis (11, 12). Our results demonstrated that RBM15B knockdown downregulated the expression of mesenchymal markers (N-cadherin and vimentin) but upregulated that of E-cadherin. These results indicate that RBM15B can enhance the migration and invasion of HCC cells by promoting EMT (**Figure S3A**). However, we did not find cell cycle changes after RBM15B knockdown in HCC-LM3 cell and Huh-7 cell (**Figure S3B**).

### RBM15B Significantly Increases HCC Resistance to Sorafenib

**Figure 3:**
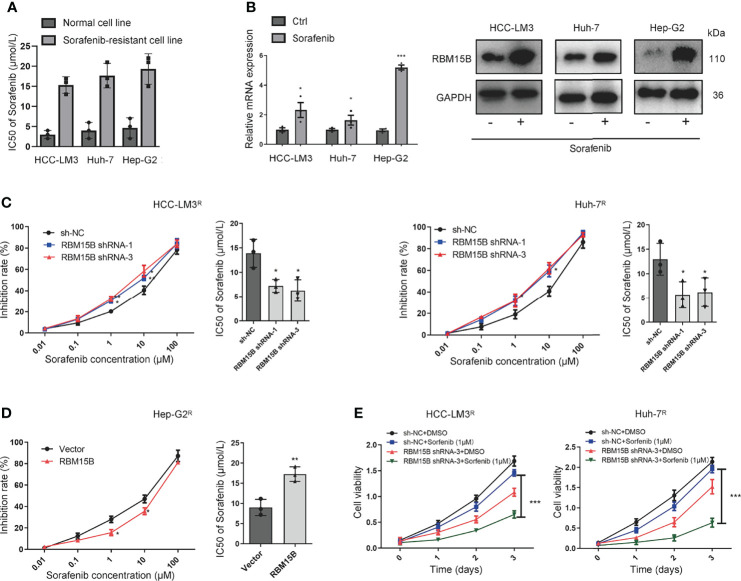
RBM15B increases HCC resistance to sorafenib. **(A)** The IC50 values in different cell lines were shown. **(B)** Human HCC cell lines were treated with sorafenib, and the protein expression of RBM15B was assayed by qRT-PCR and Western blotting. **(C)** The effect of RBM15B knockdown on sorafenib resistance. **(D)** The effect of RBM15B overexpression on sorafenib resistance. **(E)** CCK-8 assay results showing the proliferation ability of HCC cells. *p < 0.05; **p < 0.01; ***p < 0.001.

The sorafenib-resistant cell line was constructed as previously reported (13) Briefly, continuous culture of hepatoma cell lines in complete medium containing serially increasing concentrations of sorafenib to induce sorafenib resistance in cell lines. Then the half-maximal inhibitory concentration (IC50 value) was detected by the CCK-8 method (**Figure 3A**). Interestingly, we found that the expression of RBM15B was significantly upregulated in sorafenib-resistant cell lines (**Figure 3B**). These results suggested that RBM15B overexpression might be related to sorafenib resistance in HCC. According to previous reports, m6A methylation can regulate sorafenib resistance in HCC (14). Therefore, we next evaluated the relationship between RBM15B and sorafenib resistance. RBM15B knockdown increased the inhibition rate of sorafenib in the sorafenib-resistant HCC-LM3^R^ and Huh-7^R^ cell lines and decreased IC50 value, while in Hep-G2^R^ cells overexpressing RBM15B, the inhibition rate was lower, and the IC50 value was higher (**Figures 3C, D**). Our results also showed that the combination of RBM15B inhibitor and sorafenib could significantly reduce the proliferation of HCC compared with sorafenib or RBM15B inhibitor alone (**Figure 3E**). In summary, these results indicate that RBM15B promotes sorafenib resistance in HCC and provide new ideas for developing targeted drugs in the future.

### TRAM2 Was Identified as a Potential Downstream Target of RBM15B

**Figure 4:**
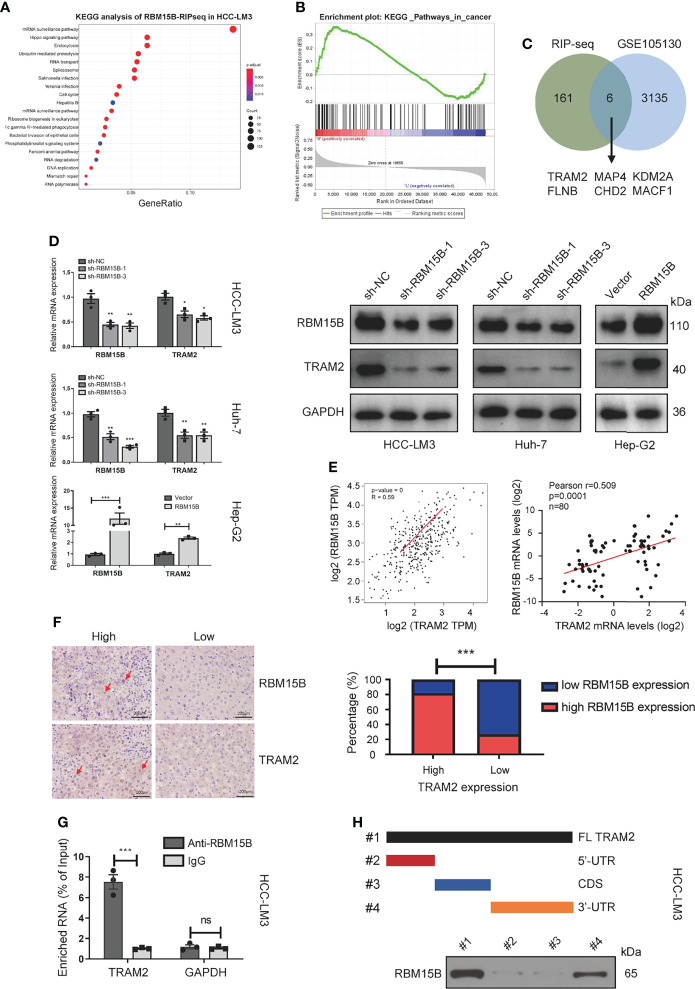
TRAM2 was identified as a potential target regulated by RBM15B. **(A)** KEGG analysis of the RIP-seq data. **(B)** A GEO dataset (GSE105130) was used for GSEA. **(C)** Identification of RBM15B-related genes through a joint analysis of multiple databases. **(D)** Detection of TRAM2 expression in Huh-7 cells with stable RBM15B knockdown. **(E, F)** Correlation between RBM15B and TRAM2 expression levels. **(G)** RIP-qPCR results showing the correlation between RBM15B and TRAM2 mRNA levels. **(H)** Schematic representation of TRAM2 mRNA depicting fragments used for biotin pull-down assays. *p < 0.05; **p < 0.01; ***p < 0.001. ns means no significant difference.

It has been reported that RBM15B is an RNA-binding protein (RBP) that recruits the m6A methylation complex to m6A sites in mRNA directly bound by RBM15B and induces the methylation of adenosine nucleotides in m6A consensus motifs (9). To clarify the downstream molecules directly bound by RBM15B, we conducted RNA immunoprecipitation-sequencing (RIP-seq) analysis. Kyoto Encyclopedia of Genes and Genomes (KEGG) analysis showed that RBM15B was significantly related to the “Hippo signaling pathway” and the “mRNA surveillance pathway”, indicating that RBM15B might bind specific Hippo signaling pathway-related mRNAs to exert a cancer-promoting effect (**Figure 4A**). We then performed gene set enrichment analysis (GSEA) on a publicly available dataset from the Gene Expression Omnibus (GEO; dataset no. GSE105130). GSEA demonstrated the enrichment of pathways related to cancer between the RBM15B high and the low groups (**Figure 4B**). We compared the genes coexpressed with RBM15B in the GES1101 dataset (R> 0.3, P <0.05) and the RIP-seq data, and we screened six genes significantly related to RBM15B and directly affected by RBM15B (**Figure 4C**). The quantitative reverse transcription-polymerase chain reaction (qRT-PCR) results showed that TRAM2 changed most significantly after knockdown or overexpression of RBM15B (**Figures S4A–C**). TRAM2 has been reported to be significantly related to the Hippo signaling pathway (15). Therefore, we identified TRAM2 as a potential downstream target of RBM15B for further research. The qRT-PCR and Western blotting results showed that TRAM2 expression decreased significantly after RBM15B knockdown and that TRAM2 expression significantly increased after RBM15B overexpression (**Figure 4D**). Based on bioinformatics analysis of the TCGA database, there was a significant positive correlation between RBM15B and TRAM2 (**Figure 4E**). In addition, we assessed the expression of RBM15B and TRAM2 in 80 pairs of HCC tissue samples. Scatter plot analysis showed that the mRNA level of RBM15B was significantly positively correlated with that of TRAM2 (R=0.529, P=0.0013; **Figure 4E**). The correlation of their protein levels was verified by immunohistochemistry (IHC) (**Figure 4F**). Finally, we verified the interaction between RBM15B and TRAM2 mRNA by RNA immunoprecipitation following quantitative polymerase chain reaction (RIP-qPCR). Compared with the IgG group, the anti-RBM15B antibody group showed significant enrichment of TRAM2 mRNA (**Figure 4G**). To map the interaction region between RBM15B and TRAM2 mRNA, various truncated forms of TRAM2 mRNA were generated. RNA pull-down analysis showed that RBM15B interacted with the 3’-untranslated region (UTR) of TRAM2 (**Figure 4H**).

### RBM15B Regulates TRAM2 mRNA Stability in an m6A-Dependent Manner

**Figure 5:**
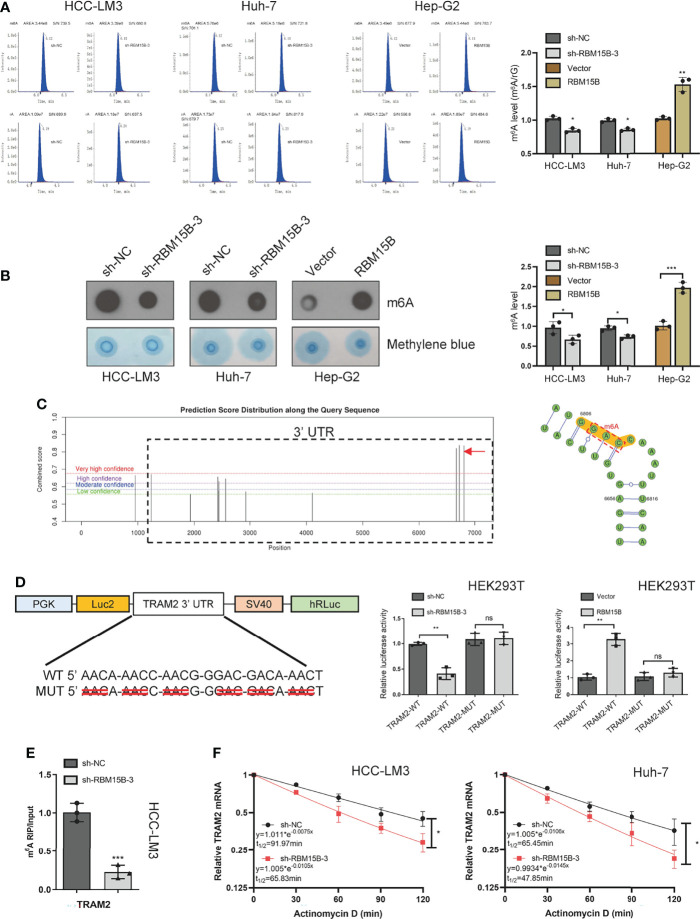
RBM15B-mediated stabilization of TRAM2 mRNA relies on RBM15B methyltransferase activity and m6A modifications in the 3’-UTR of TRAM2 mRNA. **(A)** LC/MS results showing the m6A methylation level. **(B)** Dot blot showing changes in m6A methylation. Methylene blue staining was used as a control. **(C)** The SRAMP database predicted the distribution of m6A sites in TRAM2 mRNA. **(D)** Plasmids with either WT or MUT m6A sites were constructed. The plasmid was transfected into HEK293T cells. Firefly luciferase activity was measured and normalized to Renilla luciferase activity. **(E)** m6A-RIP and qRT-PCR analysis of m6A levels in TRAM2 mRNA in HCC cell lines. **(F)** Changes in TRAM2 mRNA stability after ACT-d treatment of Huh-7 cells. *p < 0.05; **p < 0.01; ***p < 0.001. ns means no significant difference.

Although the m6A peaks were distributed in the 3’-UTR, coding sequence (CDS), 5’-UTR, and stop codon, the 3’-UTR and near stop codon region were particularly enriched (16). Based on the methyltransferase characteristics of RBM15B, we speculate that RBM15B regulates TRAM2 in an m6A-dependent manner. We first assessed whether RBM15B regulates m6A methylation in HCC. Liquid chromatography-tandem mass spectrometry (LC-MS/MS) and dot blot analysis showed that RBM15B knockdown significantly reduced the m6A level of HCC, while RBM15B overexpression induced the opposite effect (**Figures 5A, B**). To clarify the role of m6A modification in the regulation of TRAM2 mRNA, we predicted the m6A site of TRAM2 through the SRAMP database and the m6Avar database, and the results showed that there were multiple prominent m6A sites at the 3’-UTR end of TRAM2 (**Figure 5C**). Subsequently, we mutated the preferential m6A site of the TRAM2 3’-UTR (AAC, GAC) to construct a mutant (MUT) 3’-UTR luciferase plasmid and constructed a wild-type (WT) 3’-UTR luciferase plasmid for the dual-luciferase reporter assay. The luciferase activity of the TRAM2‐WT 3’-UTR was shown to be decreased after RBM15B silencing in the dual-luciferase reporter assay results, and there was no change in the luciferase activity of the RBM15B‐MUT 3’-UTR in HEK293 cells. As expected, overexpression of RBM15B increased the luciferase activity of the TRAM2-WT 3’-UTR but did not affect the luciferase activity of the TRAM2-MUT 3’-UTR in HEK293 cells (**Figure 5D**). Importantly, through the m6A-RIP assay, we found that the m6A antibody significantly enriched the 3’-UTR of TRAM2 mRNA and that RBM15B knockdown reduced the m6A level on the 3’-UTR of TRAM2 mRNA (**Figure 5E**). Moreover, we used actinomycin D (ACT-d) to inhibit transcription in Huh-7 and HCC-LM3 cell lines transduced with sh-NC and sh-RBM15B-3 and measured the relative changes in TRAM2 mRNA levels over time (**Figure 5F**). Our results showed that RBM15B knockdown significantly decreased the stability of TRAM2 mRNA. These results indicated that RBM15B enhances the stability of TRAM2 mRNA and that this process is dependent on the m6A methyltransferase activity of RBM15B in the TRAM2 3’-UTR. METTL3 recruits YTHDF1 to regulate mRNA stability (17). We tried to clarify whether the m6A modification of TRAM2 by RBM15B depends on YTHDF1. However our results showed that YTHDF1 knockdown did not affect the stability of TRAM2 mRNA (results not shown).

### TRAM2 Promotes the Proliferation and Invasion of HCC

Studies have shown that TRAM2 promotes the proliferation and invasion of oral and breast cancer cells, but no reports have demonstrated the role of TRAM2 in HCC cells (15, 18). We used siRNA to construct TRAM2 knockdown models in Huh-7 and HCC-LM3 cell lines (**Figure S5A**). The results of the CCK-8 assay and colony formation assay revealed that TRAM2 knockdown inhibited the proliferation of HCC cells (**Figures S5B, C**). In addition, scratch wound healing motility assays and Transwell invasion assays showed that TRAM2 knockdown significantly reduced cell invasion and migration (**Figures S5D, E**). Furthermore, we examined the relationship between TRAM2 and sorafenib resistance in HCC. TRAM2 knockdown increased the inhibition rate of sorafenib in the sorafenib-resistant HCC cell lines and decreased the IC50 value (**Figure S5F**). It has been reported that TRAM2 is recognized as key mediator of YAP-induced oncogenic cellular activities, such as cell proliferation and invasiveness (15). Therefore, we examined YAP/TAZ the protein level changes and phosphorylation level changes after TRAM2 knockdown. Our results showed that the expression and phosphorylation levels of YAP and TAZ were significantly reduced after TRAM2 knockdown, suggesting that TRAM2 knockdown inhibited the expression and activation of YAP and TAZ, thereby inhibiting the Hippo signaling pathway (**Figure S5G**).

### RBM15B Promotes HCC Growth and Metastasis Through TRAM2

**Figure 6:**
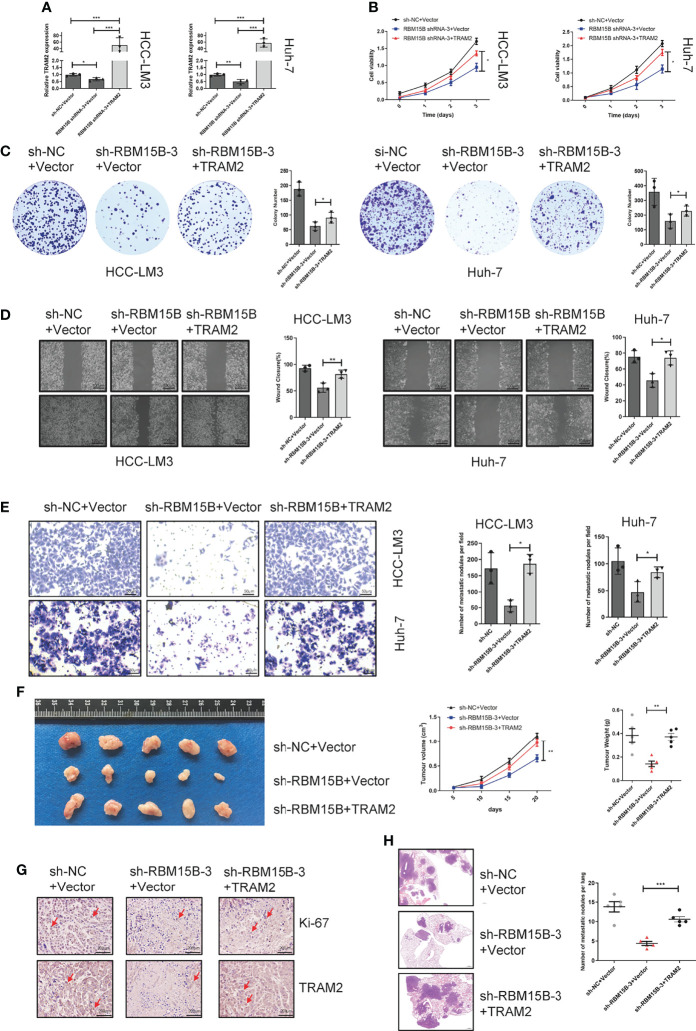
TRAM2 is an important downstream target of RBM15B. **(A)** Detection of TRAM2 mRNA expression in different groups of HCC cell lines. **(B, C)** CCK-8 and colony formation assay results showing the proliferation ability of HCC cell lines. **(D)** Scratch wound healing motility assay results showing the migration of HCC cell lines. **(E)** Transwell Matrigel invasion assay showing the invasion of HCC cell lines. **(F)** Assessment of the size and weight of the dissected tumors after 5 weeks. **(G)** TRAM2 and Ki-67 expression was determined by IHC analysis. **(H)** Metastasis in the lungs of nude mice injected with different HCC cells. *p < 0.05; **p < 0.01; ***p < 0.001.

To further assess whether the oncogenic effect of RBM15B is dependent on TRAM2, we conducted a rescue experiment as follows: we induced the overexpression of TRAM2 in cells with stable RBM15B knockdown (**Figure 6A**). The CCK-8 and colony formation assay showed that overexpression of TRAM2 significantly reversed the inhibitory effect of RBM15B knockdown on cell proliferation (**Figures 6B, C**). In addition, scratch wound healing motility assays and Transwell invasion assays showed that TRAM2 expression reversed the suppression of migration and invasion caused by RBM15B silencing (**Figures 6D, E**). We also performed the rescue experiment *in vivo*. Overexpression of TRAM2 attenuated the reduction in subcutaneous tumor volume in nude mice caused by knockdown of RBM15B (**Figure 6F**). Ki‐67 staining showed that the sh-RBM15B+TRAM2 group had significantly higher levels of Ki‐67-positive cells than the sh-RBM15B+Vector group (**Figure 6G**). The tail vein lung metastasis assay showed that TRAM2 overexpression corresponded to an increased number of lung metastatic nodules (**Figure 6H**). These findings strongly suggested that RBM15B mediates the proliferation and metastasis of HCC cells at least partially by regulating the expression of TRAM2.

### YY1 Promotes the Transcription of RBM15B

**Figure 7:**
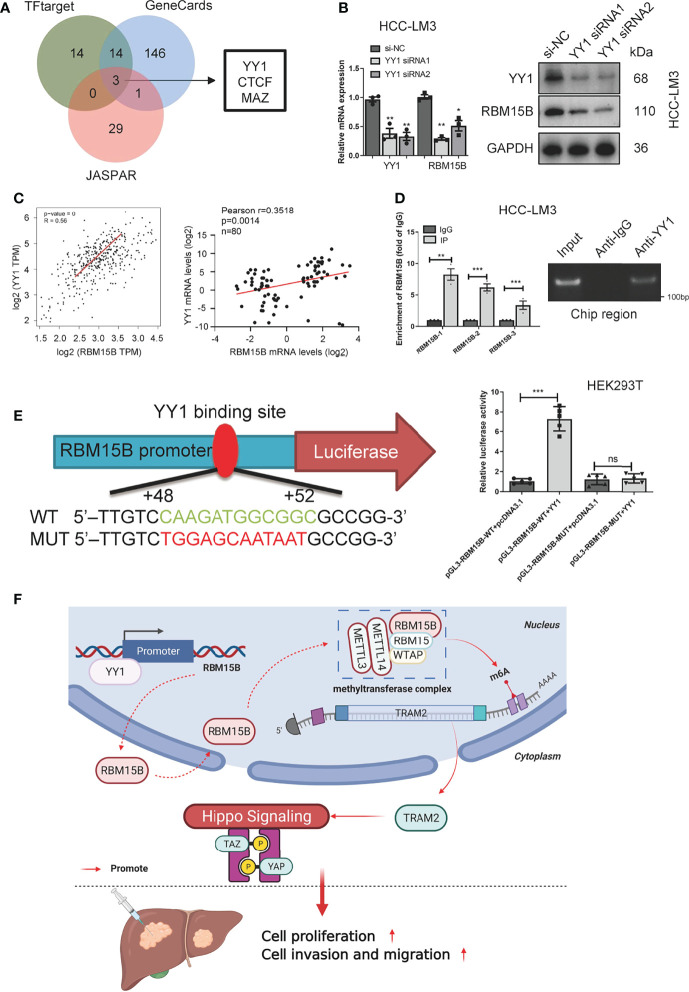
RBM15B is directly regulated by the transcription factor YY1. **(A)** Joint analysis of multiple databases predicted YY1 as a transcription factor of RBM15B. **(B)** After YY1 knockdown, RBM15B mRNA and protein levels were significantly reduced. **(C)** Pearson correlation analysis of RBM15B and YY1 in the TCGA-LIHC dataset and HCC tissue samples. **(D)** ChIP results showed that YY1 binds to RBM15B. **(E)** YY1 binding sites in the WT and MUT RBM15B promoters. Dual-luciferase reporter assays showed that YY1 acts on the RBM15B promoter region. **(F)** Schematic diagram of the RBM15B signaling pathway. *p < 0.05; **p < 0.01; ***p < 0.001. ns means no significant difference.

To clarify the reason for the overexpression of RBM15B in HCC, we predicted and screened the upstream transcription factors of RBM15B through the GeneCards database, hTF target database and JASPAR database (**Figure 7A**). The qRT-PCR results showed that YY1 knockdown significantly reduced the expression of RBM15B in HCC cells (**Figure 7B**). In addition, our results showed that the RBM15B mRNA level was not affected by selective knockdown of CTCF or MAZ (results not shown). YY1 was identified as the main transcription factor of RBM15B and was selected for further study due to its active role in the proliferation and invasion of HCC (19). The TCGA database correlation analysis results showed that YY1 was positively correlated with RBM15B (**Figure 7C**). In addition, we observed a significant positive correlation between the mRNA levels of YY1 and RBM15B in 80 pairs of HCC tissues (r=0.3518, P=0.014; **Figure 7C**). Subsequently, we performed a chromatin immunoprecipitation (ChIP) assay, and the results confirmed that YY1 binds to the RBM15B promoter region (**Figure 7D**). We constructed a HEK293T cell line containing a luciferase reporter vector containing the WT or MUT YY1 binding sequence. The dual-luciferase reporter gene test results showed that the YY1 binding site mutation eliminated the ability of YY1 to promote the transcription of RBM15B (**Figure 7E**). In summary, our results indicate that the transcription factor YY1 promotes RBM15B transcription by binding to the RBM15B promoter region. Furthermore, we examined the relationship between YY1 and sorafenib resistance in HCC. Unfortunately, we have not found that YY1 affects sorafenib resistance in HCC (**Figure S6**).

## Discussion

m6A is the primary internal epigenetic modification in eukaryotic mRNA and is similar to DNA methylation and protein phosphorylation (8, 17). The discovery of m6A facilitated the birth of epitranscriptomics, and m6A modification has been found to have a wide range of important biological effects. The process of RNA modification is dynamic and reversible, and changing m6A levels affects RNA processing, degradation and translation, which affect gene expression and critical cellular processes and ultimately lead to tumorigenesis and disease progression (20). Among the factors involved in this process, m6A writers (METTL3, METTL14, KIAA1429, WTAP, RBM15, RBM15B and ZC3H13) can catalyse the installation of m6A on RNA (21). m6A erasers (FTO and ALKBH5) can delete these modifications. Finally, readers (YTHDC1, YTHDC2, YTHDF1, YTHDF2 and HNRNPC) can recognize m6A methylation and affect mRNA splicing and stability, degradation, translation and other biological processes (7). Interestingly, our results showed that as a part of the MTC, RBM15B also has some functions of readers, which implies that the boundary between writers and readers is not absolute. This is consistent with a previous report that RBM15/RBM15B can link the methylation complex to specific RNA (9). Although m6A and its related regulatory factors are involved in the pathogenesis of many diseases, including cancer, and may provide new targets for cancer treatment, there is still a limited understanding of the underlying mechanism of m6A modification (22).

RBM15B, METTL3 and WTAP together form the MTC, which catalyses the installation of m6A to a specific RNA site and exerts its m6A modification effect. The role of m6A methyltransferases in tumors has been partially reported; for example, METTL3 upregulation promotes the malignant progression of HCC by inhibiting SOCS2 expression through an m6A-dependent mechanism, and METTL14 inhibits proliferation and metastasis in colorectal cancer. However, no reports have mentioned whether RBM15B plays a role in tumorigenesis. Our study showed for the first time that RBM15B could predict a poor prognosis in HCC patients and was significantly related to tumor size, TNM stage and PVTT stage. In the current study, we showed that RBM15B was overexpressed in HCC and promoted the proliferation, invasion and EMT transition of HCC cells. RBM15B overexpression significantly upregulated the level of m6A in HCC. It has been reported that m6A modification plays an essential role in regulating tumor drug resistance (14). For example, m6A methylation regulates sorafenib resistance in liver cancer through FOXO3-mediated autophagy, and m6A-modified circRNA-SORE sustains sorafenib resistance in HCC by regulating β-catenin signaling (14, 23). Our results showed that RBM15B and TRAM2 knockdown significantly inhibited HCC resistance to sorafenib, while overexpression had the opposite effects. In addition, our results showed that RBM15B could improve the therapeutic effect of sorafenib, which suggests the possibility of combining RBM15B inhibitors and sorafenib.

Subsequent RIP-seq data and gene coexpression data were jointly analysed, and TRAM2 was identified as a downstream target of RBM15B. According to reports, the correct splicing of mRNA requires m6A modification, the disruption of which will lead to the early degradation of mRNA; for example, IGF2BP proteins enhance mRNA stability, and the recognition of m6A by YTHDF1 results in enhanced protein synthesis (16, 24). However, these divisions are not absolute. Several neurotransmitters have been reported to reduce m6A mRNA stability. For example, the m6A reader protein YTHDF2 has been previously reported to promote mRNA degradation by recognizing m6A and recruiting the mRNA decay machinery (25, 26). Our results showed that RBM15B bound to TRAM2 3’-TUR and increased the stability of TRAM2 mRNA in a m6A-dependent manner, which might be a significant cause of tumorigenesis and progression. TRAM2 is a crucial determinant of cancer biology, including cell proliferation and chemotherapeutic drug resistance, and has a significant impact on prognosis. TRAM2 can activate YAP to promote tumor growth and metastasis, but no research has been conducted to clarify the function of TRAM2 in liver cancer (15, 18). We reported the function of TRAM2 in HCC for the first time, and our results showed that TRAM2 promoted HCC cell proliferation and metastasis. Subsequent functional recovery experiments showed that TRAM2 overexpression rescued the inhibitory effect of RBM15B on HCC cell proliferation and metastasis. Although TRAM2 may not be the only target gene of RBM15B, our results confirmed that TRAM2 is an essential downstream target of RBM15B in HCC regulation.

YY1 is a transcription factor that is highly expressed in many cancers and is related to cell proliferation, survival and metabolic reprogramming (19, 27). YY1 is known to regulate the transcriptional activation and inhibition of many genes related to various cellular processes, including cell differentiation, DNA repair, autophagy, cell survival and apoptosis, and cell division (28, 29). It is considered to be the main driving factor for many cancers (19). Our research shows that YY1 can directly bind to the promoter region of RBM15B to activate the transcription of RBM15B, and in clinical samples, it has a significant positive correlation with RBM15B.

The m6A modification is a double-edged sword in cancer. It plays a role in promoting or suppressing cancer by regulating the expression levels of different genes. Our results reveal a new regulatory mechanism; specifically, RBM15B is activated by YY1 for transcription and regulates the stability of TRAM2 mRNA in an m6A-dependent manner, thereby promoting the proliferation and metastasis of HCC (**Figure 7F**). These results are significant for the development of targeted cancer therapies related to m6A. However, the role of RBM15B in other tumors and the particular preference of downstream genes need to be further explored.

## Materials and Methods

### Clinical Specimens

This study was approved by the Ethics Committee of Zhongnan Hospital of Wuhan University (2020032). This study included eighty pairs of HCC and nontumor specimens from liver cancer patients who underwent surgery at Zhongnan Hospital of Wuhan University between 2015 and 2020. Tissue samples were collected within 30 minutes after surgery and stored in liquid nitrogen tanks. HCC tissue was confirmed by using IHC by two blinded pathologists. None of the patients received radiotherapy or chemotherapy before surgery, and all patients signed informed consent forms. In our study, HCC patients were diagnosed based on the American Association for the Study of Liver Diseases (AASLD) practice guidelines.

### Cell Culture

Human liver cancer cells (HCC-LM3, Huh7, Hep-G2, Hep-G2.215, SK-Hep1 and 7721) were purchased from the Shanghai Cell Bank of the Chinese Academy of Sciences. The human liver cell line HL-7702 (L02) was purchased from Wuhan Hengyisai Biotechnology Co., Ltd. Short tandem repeat (STR) profiling was used to identify all cells. All cells were cultured in an incubator at 37°C and 5% carbon dioxide using DMEM with 10% FBS.

### Immunochemistry and Immunofluorescence

Immunohistochemistry and immunofluorescence were performed as described previously (30). The IHC-stained tissue sections were scored by two pathologists who were blinded to the clinical parameters. The scoring for staining intensity was as follows: 0 (negative staining), 1 (weak), 2 (medium), 3 (strong). The final expression score is intensity score × extent score. Those with a total score of more than 6 points were regarded as the high expression group, and the rest were regarded as the low expression group (**Figure S7**).

### Quantitative Real-Time PCR (qRT-PCR)

TRIzol reagent was used to extract total RNA from HCC tissues and cells. Reverse transcription of RNA into cDNA was performed using a kit according to the manufacturer’s instructions. SYBR Green mix was used to perform qRT-PCR to evaluate the transcription level of target genes. All primer sequences are provided in **Supplementary Table S1**.

### Western Blotting

Western blotting was performed as described previously (30). All antibody information is shown in **Supplementary Table S2**.

### Plasmid, Lentiviral Construction and Cell Transfections

The plasmids were designed and synthesized by GenePharma Company (Shanghai, China). The siRNA was designed and constructed by Wuhan Biological Company (Wuhan, China). According to the manufacturer’s protocol, siRNA was transfected using Genmute reagent (SignaGen Laboratories, MD, USA). Lipo3000 (Invitrogen, Waltham, Massachusetts, USA) was used for plasmid transfection according to the manufacturer′s instructions. The lentiviruses carrying shRNA were packaged by Gene Create Company (Wuhan, China). HCC cells were transduced with a lentivirus. After 48 h of infection, puromycin (2 μg/mL) was added to the medium to select positively infected cells. The siRNA and shRNA sequences are shown in **Supplementary Table S3**.

### CCK-8 Assay

Cell proliferation was detected using cell counting kit-8 (CCK-8). To perform the CCK-8 assay, HCC cells were plated at 2x10^4^ cells per well in 96-well plates. Ten microlitres of CCK-8 reagent (Beyotime Biotechnology, Shanghai, China) was added and incubated for another 2 hours at 37°C. Optical density (OD) was measured at 450 nm with a microplate reader in the next step.

### Clone Formation Assay

Cell proliferation was detected using the colony formation assay. Cells were seeded into 6-well plates at a density of 1000 cells/well. The media were changed every two days, and the cells were allowed to grow for 14 days. Cells were fixed with 4% paraformaldehyde and stained with 1% crystal violet. Cell colonies containing at least 20 cells were counted using an inverted microscope at 40× magnification.

### Scratch Wound-Healing Motility Assay

Cell migration was determined using a scratch wound-healing motility assay. HCC cells were suspended in DMEM without FBS and seeded at a density of 1×10^6^ cells per well in 6-well culture plates. Wounds were created using a 200-μl plastic pipette tip. Photographs were captured at 0 and 24 h and were analysed with ImageJ software to calculate the scratched areas.

### Transwell Invasion Assay

Cell invasion was determined using a Transwell invasion assay. In brief, the experiment was performed using Transwell chambers (8-μm pore size; Corning) in 24 healthy plates. In the serum-free medium in the upper chamber, 1×10^5^ cells were sown; however, the medium with 10% FBS was placed in the lower chamber. After incubation for 48 h, the cells in the upper chamber were fixed with methanol and stained with 0.1% crystal violet. Cells on the upper side of the Transwell membrane were wiped off with a cotton swab, and cells on the underside of the Transwell membrane were photographed and quantified under a microscope.

### *In Vivo* Experiments

The Wuhan University of Zhongnan Hospital Ethics Committee approved the animal study. Male athymic 5-week-old BALB/c nude mice were purchased from Wuhan University Medical Research Center. The mice were randomly divided into two groups and kept in a specific-pathogen-free (SPF) environment, and the researchers were unaware of the grouping. No statistical methods were used to predetermine the sample size. HCC-LM3 cells transfected with sh-NC and sh-RBM15B-3 were injected into the axilla or tail vein of mice. Tumor size was measured with a Vernier calliper every five days. After 20 days, the mice were humanely sacrificed in accordance with the National Institutes of Health Laboratory Animal Care and Use Guidelines. The tumors and lung lobes were photographed and weighed.

### RIP-Seq

For immunoprecipitation, the supernatant was incubated with 10 μg of Flag antibody and control IgG antibody at 4°C overnight. The immunoprecipitate was incubated with Protein A/G Dynabeads for an additional 2 h at 4°C, and the beads were then washed. Proteinase K (Roche) was added to 1% input, and RBPs were immunoprecipitated with cross-linked RNA to a final concentration of 1.2 mg/ml. The samples were incubated at 55°C for 120 minutes. RNA was purified with TRIzol reagent (Life Technologies). The cDNA library was generated using the Illumina ScriptSeq™ v2 RNA-Seq Library Preparation Kit (Epicentre). For high-throughput sequencing, the libraries were prepared following the manufacturer’s instructions and applied to the Illumina NextSeq500 system for 150 nt paired-end sequencing by ABlife, Inc. (Wuhan, China).

### The Dual-Luciferase Assay

The dual-luciferase assay was performed according to our previously reported protocols (31).

### ChIP Assay

ChIP assay was performed as detailed previously (31).

### Dot Blot Assay

The RNA sample was spotted on the nylon membrane, and UV cross-linking was performed. Then, 10 mL of PBST containing 5% BSA was added to seal the membrane. Subsequently, the m6A antibody was used for incubation, and the enhanced chemiluminescence (ECL) system (Tanon, USA) was used to detect the signal. Finally, methylene blue staining was used as an RNA loading control.

### LC-MS/MS Quantification of m6A

The nucleoside mixture after the RNA guy was extracted with chloroform. The resulting aqueous layer was collected and used for liquid chromatography-electrospray ionization-tandem mass spectrometry (LC-ESI-MS/MS) (UPLC, ExionLC AD; MS, Applied Biosystems 6500 Triple Quadruole) analysis.

### Methylated RNA Immunoprecipitation (Me-RIP) Assay

The RNA was fragmented into approximately 100 nucleotides and then purified using the RNase MiniElute kit. A/G magnetic beads and m6A antibody were used for specific enrichment. After elution, reverse transcription and amplification, qPCR analysis was performed on the m6A-IP and IgG-IP components, and the corresponding threshold cycle (CT) value was calculated.

### RIP-qPCR Assay

The RIP-qPCR assay was performed as detailed previously (32).

### RNA Pull‐Down Assay

RNA pull‐down assays were performed as described previously (32).

### Actinomycin D Chase Experiment

Actinomycin D (Act D) chase experiments were performed for mRNA stability assays. Briefly, cells were incubated with 10 μg/ml actinomycin D (Act-D, Catalogue #A9415, Sigma, St. Louis, MO, USA) diluted in DMEM for 0, 1, 2 and 4 h. RNA was extracted at each time point using TRIzol Reagent (Takara), and mRNA expression was evaluated by qRT-PCR analysis.

### Statistical Analysis

GraphPad Prism 8.0 software and SPSS 21.0 software were used for statistical analysis. Student’s t-test and the χ2 test were used as appropriate for statistical analysis of the parameters. The overall survival rate was calculated according to Kaplan-Meier analysis. All data are the results of three independently repeated experiments. For all tests, a P-value of <0.05 was considered statistically significant.

## Data Availability Statement

The datasets presented in this study can be found in online repositories. The names of the repository/repositories and accession number(s) can be found in the article/**Supplementary Material**.

## Ethics Statement

The studies involving human participants were reviewed and approved by Zhongnan Hospital of Wuhan University’s Protection of Human Subjects Committee. The patients/participants provided their written informed consent to participate in this study. The animal study was reviewed and approved by the Laboratory Animal Ethics Committee, Zhongnan Hospital of Wuhan University (Hubei province, China).

## Author Contributions

ZL conceived and designed the experiments. PX and CT carried out the major part of the project. HZ collected and analyzed the clinical data. CT and PX wrote the manuscript. DG, KX, and PL constructed a variety of animal models. ZL provided the funding and contributed to the critical review of the manuscript. All authors read and approved the final manuscript.

## Funding

This work was supported by grant from the National Natural Science Foundation of China (81772926) and Cancer research and translational platform project of Zhongnan Hospital of Wuhan University (ZLYNXM202004).

## Conflict of Interest

The authors declare that the research was conducted in the absence of any commercial or financial relationships that could be construed as a potential conflict of interest.

## Publisher’s Note

All claims expressed in this article are solely those of the authors and do not necessarily represent those of their affiliated organizations, or those of the publisher, the editors and the reviewers. Any product that may be evaluated in this article, or claim that may be made by its manufacturer, is not guaranteed or endorsed by the publisher.
