# Supplementary material for: YY1-Targeted RBM15B Promotes Hepatocellular Carcinoma Cell Proliferation and Sorafenib Resistance by Promoting TRAM2 Expression in an m6A-Dependent Manner
Source: Front Oncol. 2022 Apr 14;12:873020. doi: 10.3389/fonc.2022.873020 (PMC9046568; doi:10.3389/fonc.2022.873020)
Supplement: Supplementary file 1 [file DataSheet_1.pdf]

A

B

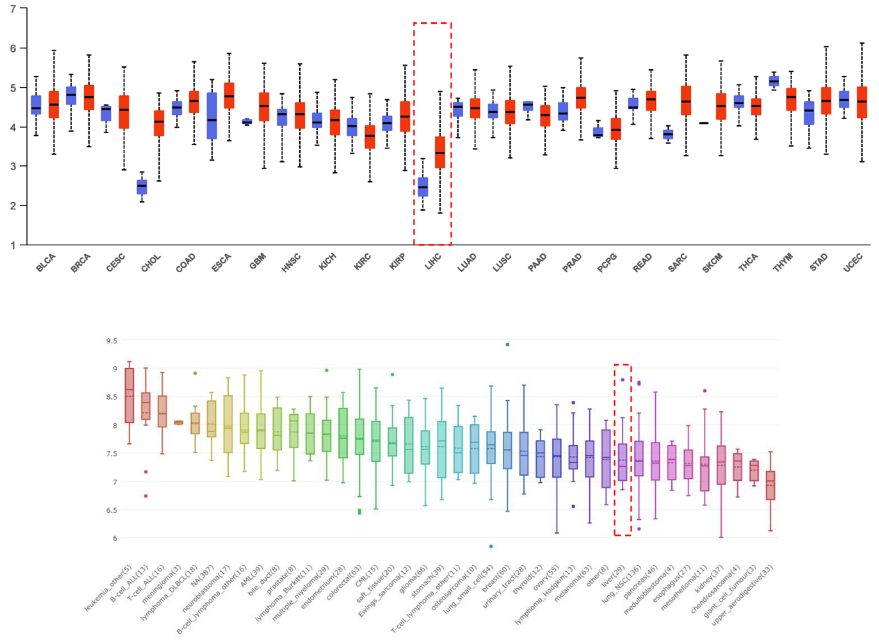

Fig. S1 The mRNA expression analysis of RBM15B

A Analysis of RBM15B mRNA expression according to the TCGA database. B Analysis of RBM15B mRNA expression according to the CCLE database.

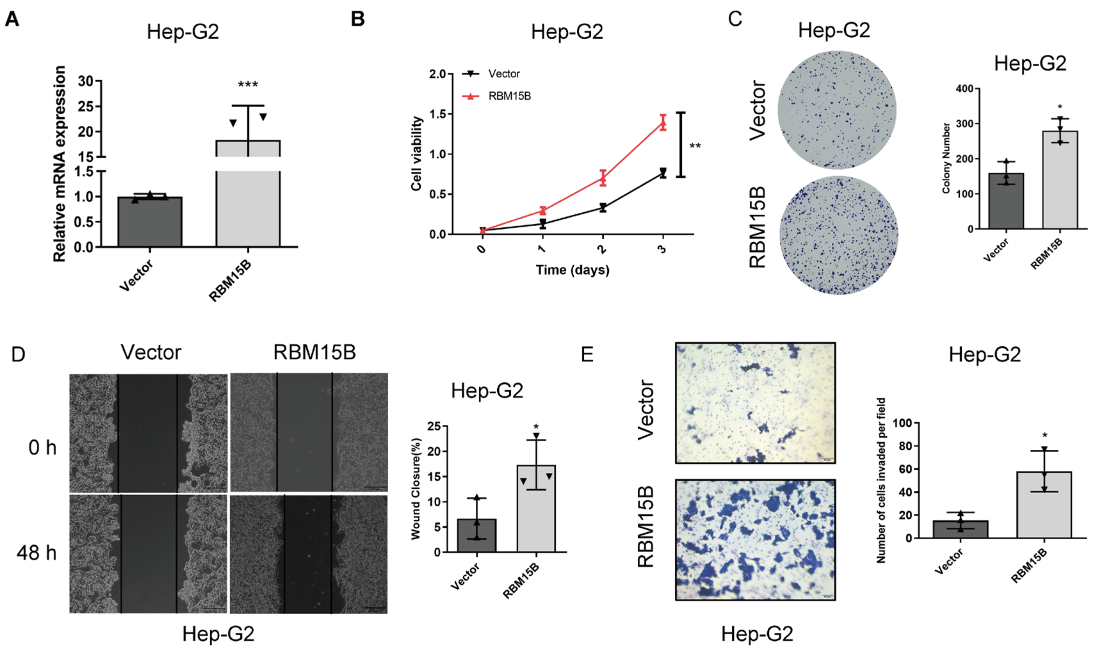

Fig. S2 RBM15B overexpression promotes HCC cell growth and metastasis

A The RBM15B-overexpressing plasmid was transformed into the Hep-G2 cell line. B, C CCK-8 and colony formation experiments showed that RBM15B functions in cell proliferation. D, E The effect of RBM15B overexpression on cell invasion and migration.

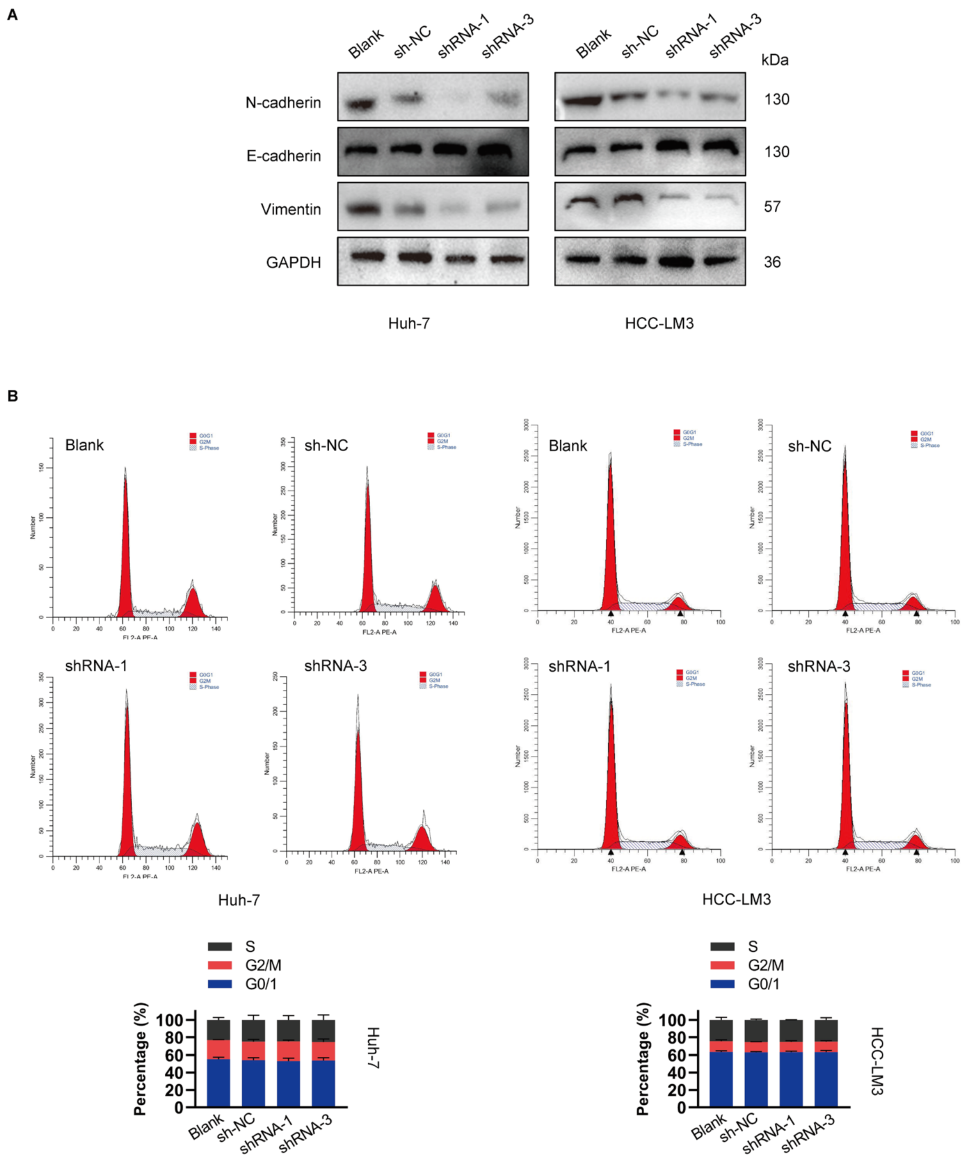

Fig. S3 RBM15B promotes epithelial-mesenchymal transition (EMT)

A Western blot results showed the effect of RBM15B knockdown on EMT in Huh-7 and HCC-LM3 cells. B Flow cytometry results showed the effect of RBM15B knockdown on cell cycle progression.

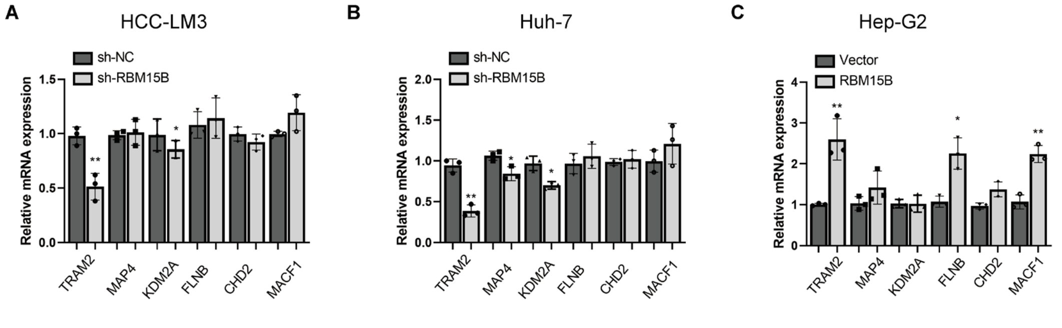

Fig. S4 Differential gene expression analysis of cells with RBM15B knockdown and overexpression

A, B Analysis of differentially expressed genes in RBM15B knockdown versus control Huh-7 and HCC-LM3 cells. C Analysis of differentially expressed genes in RBM15B-overexpressing versus control Hep-G2 cells.

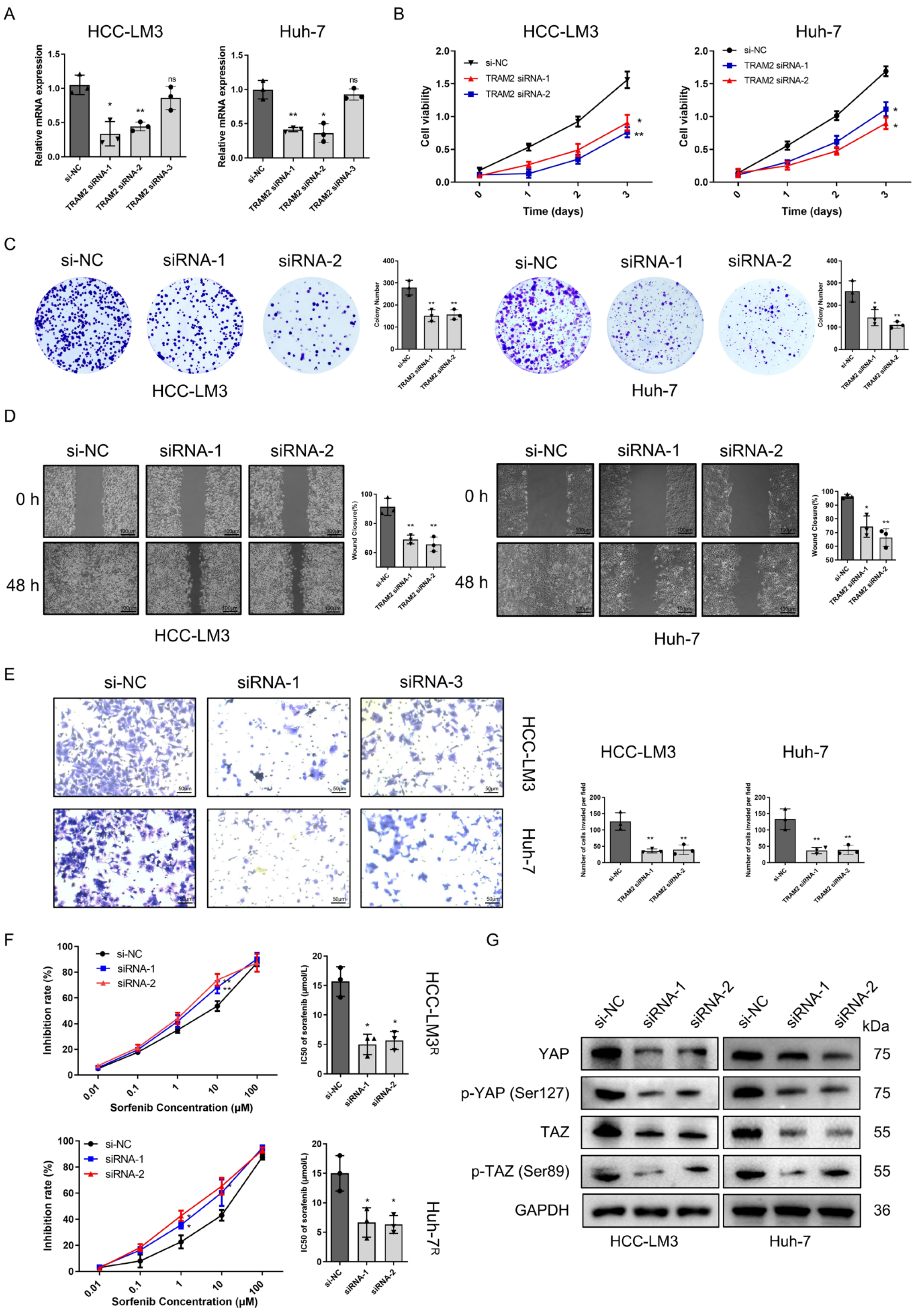

Fig. S5 TRAM2 promotes HCC cell proliferation and metastasis

A The expression of TRAM2 knockdown in HCC cell lines by siRNA. B, C CCK-8 and colony formation assays showing the proliferation ability of HCC cell lines. D, E Scratch wound healing motility assay showing cell migration in HCC cell lines. E Transwell Matrigel invasion assay showing cell invasion in HCC cell lines. F The effect of TRAM2 knockdown on sorafenib resistance. G Detection of YAP, TAZ, p-YAP, p-TAZ expression in HCC cells with TRAM2 knockdown.

A

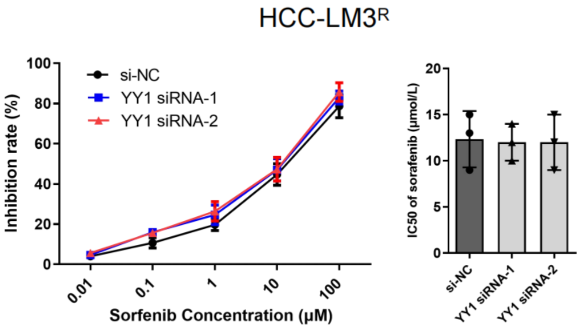

B

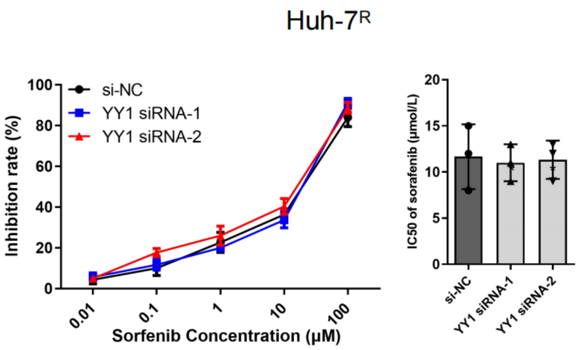

Fig. S6 The effect of YY1 on sorafenib resistance.

A, B The effect of YY1 knockdown on sorafenib resistance.

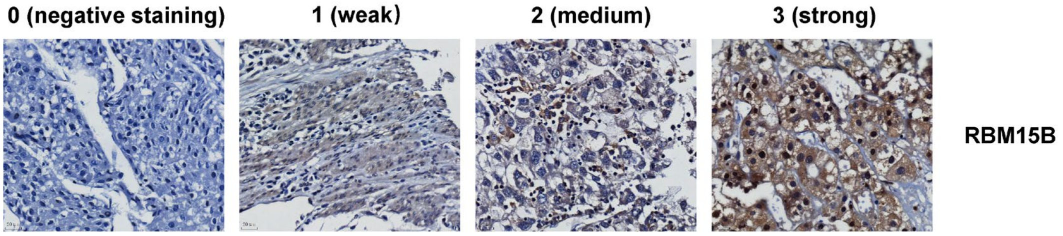

Fig. S7 Representative images of different intensities of RBM15B IHC staining.
