# Supplementary material for: YY1-Targeted RBM15B Promotes Hepatocellular Carcinoma Cell Proliferation and Sorafenib Resistance by Promoting TRAM2 Expression in an m6A-Dependent Manner
Source: Front Oncol. 2022 Apr 14;12:873020. doi: 10.3389/fonc.2022.873020 (PMC9046568; doi:10.3389/fonc.2022.873020)
Supplement: Supplementary file 2 [file DataSheet_2.pdf]

Supplementary Table S1 Primer sequences used in this study.

Primer name		Sequence (5'-3')
RBM15B	F	TGAGGAACGGAGTAGGACCAA
	R	CTCGCTGTCTCTTGCCTTCT
TRAM2	F	TTCCGCAGGAGGACGAAAAG
	R	ACCTCGAACATAAGCCCGATG
YY1	F	ACGGCTTCGAGGATCAGATTC
	R	TGACCAGCGTTTGTTCAATGT
GAPDH	F	GAAGGTGAAGGTCGGAG
	R	GAAGATGGTGATGGGATTTC
MAP4	F	TCTGAAGAATGTCCGCTCCAA
	R	TCTGAAGAATGTCCGCTCCAA
KDM2A	F	GGAGGTCTGGATGTCTGT
	R	GGAGGTCTGGATGTCTGT
FLNB	F	TCCTCCACTACTCCATCTCCAT
	R	TTGCCGTCTTGCCAGTTCT
CHD2	F	CCGAAGCAGACAAGAACCATC
	R	CCGCTACTTGCCTCTTCCTTA
MACF1	F	ACAGCAGCACATCCATACGA
	R	TAGTTCCTCCTCCTCCTTCTCA
CTCF	F	CTCATCCAGCATCAGAAGT
	R	CTCATCCAGCATCAGAAGT
MAZ	F	GCGTCTGCCTTGGAGAAGA
	R	GGAGATTGTAGCCGTTCTTGAA
RBM15B	F	GCGTGGCAAGGTGTGATAA
Primer 1	R	GCAGTTGATGGAGTTGGAATAG
RBM15B	F	TCGTCCTGAAGGTGATGAACA
Primer 2	R	CGTAAGAGCCAAGGATGAAGAG
RBM15B	F	GGTCGTCCTGAAGGTGATGA
Primer 3	R	CGTAAGAGCCAAGGATGAAGAG

Supplementary Table S2 Primary antibodies used in this study

Antigens	Manufacturer	Catalog Number	Application
RBM15B	Proteintech	67506-1-Ig	1:1000 for WB;
	Proteintech	22249-1-AP	1:500 for IHC
TRAM2	Proteintech	13311-1-AP	1:1000 for WB
N-cadherin	Proteintech	22018-1-AP	1:1000 for WB
E-cadherin	Proteintech	20874-1-AP	1:1000 for WB
Vimentin	Proteintech	10366-1-AP	1:1000 for WB
YAP	Cell Signaling Technology	14074	1:1000 for WB
TAZ	Cell Signaling Technology	72804	1:1000 for WB
p-YAP	Cell Signaling Technology	13008	1:1000 for WB
p-TAZ	Cell Signaling Technology	59971	1:1000 for WB
YY1	Proteintech	2E11C5	1:1000 for WB
GAPDH	Proteintech	1E6D9	1:1000 for WB
N6-Methylguanosine	Abbkine	ABP52775	Me-RIP
	Scientific	202003	1:500 for Dot Blot
IgG	Servicebio	GB23301	IP
HRP	IPKine	A25222	1:1000 for WB; IP

Supplementary Table S3 siRNAs and shRNA used in this study.

Primer name	Sequence (5’-3’)
sh-NC	TTCTCCGAACGTGTCACGT
sh-RBM15B-1	GGTGTTGCTTAAAGACAAA
sh-RBM15B-2	GGTGTTTGTTTGTTTGTTA
sh-RBM15B-3	AAAGCUAUCUCCUUUGACGUG
si-NC	UUCUCCGAACGUGUCACGUTT
si-TRAM2-1	GGAUCUCUCUCCAGCUCAAGG
si-TRAM2-2	GCAUCAUCUUAGAGGUCUAUU
si-TRAM2-3	AGGUAAAAUAAAUAGAAAGGC
si-CTCF-1	CAGACUCUGUAGUGUGUAAAU
si-CTCF-2	GGACAGUGUUGACAACUAACU
si-MAZ-1	GAGAAGAGAUGGAGUCUUAGG
si-MAZ-2	ACAAAUCGUUAAAACCUAGCG
si-YY1-1	GCCUCUCCUUUGUAUAUUAUUTT
si-YY1-2	GACGACGACUACAUUGAACAATT
